# Using Partner-Driven Maximum Variance Sampling to Form a Lived Experience Panel: Step-by-Step Tutorial

**DOI:** 10.2196/95145

**Published:** 2026-06-26

**Authors:** Anna Jolliff, Teresa Thuemling, Jessica Arora, Geri L Baumblatt, Daniel J Digmann, Ursulá T Garcia-Mayes, Misty Hawkins, Sydney Hoel, Heather Janes, Nicole Orendain, Alan Richmond, Aracely Rosales, Pete Wendel, Nicole E Werner, Rupa S Valdez

**Affiliations:** 1 Center for Research and Innovation in Systems Safety, Department of Anesthesiology, Vanderbilt University Medical Center, 2525 West End Avenue, Suite 800, Nashville, TN, 37203, United States, 1 615-421-4210; 2 Department of Systems and Information Engineering, School of Engineering and Applied Science, University of Virginia, Charlottesville, VA, United States; 3 Rush University Medical Center, Chicago, IL, United States; 4 Community Partner, Charlottesville, VA, United States; 5 HIS AhMayesIng Grace LLC, Duluth, GA, United States; 6 Department of Applied Health Science, School of Public Health, Indiana University, Bloomington, IN, United States; 7 Center for Health by Design, School of Public Health, Indiana University, Bloomington, IN, United States; 8 Monarch Dementia Caregiver Services, Pierre, SD, United States; 9 South Dakota Chapter, Alzheimer's Association, Pierre, SD, United States; 10 Community-Campus Partnerships for Health, Raleigh, NC, United States

**Keywords:** tutorial, patient engagement, maximum variance sampling, lived experience panel, advisory board, family caregivers

## Abstract

Research projects conducted in collaboration with patients and caregivers are more rigorous, relevant, and impactful within the communities of focus. One mechanism for facilitating patient and caregiver engagement in research is through a lived experience panel (LEP)—an intentionally assembled group of people with personal experience relevant to the research topic. LEPs can engage in any or all aspects of the research project, from study ideation to participant recruitment to dissemination and translation of findings. Fully realizing the potential of an LEP to inform the research process requires intentionality in LEP composition, as members should align with the range of lived experiences in the community of focus. Mechanisms to recruit patient or caregiver advisors for research may require researchers to reach beyond pre-existing relationships if they intend to achieve maximum variability. However, explicit processes for forming a maximally variable LEP are scarce. In this guide, we outline an LEP formation process that includes laying the foundation for recruitment with academic and community partners; collaboratively developing promotional materials and an interest survey; using community-embedded promotional channels to share information about the LEP opportunity; and selecting interviewees and LEP invitees using a process called maximum variance sampling. Outcomes of our LEP formation process include the relative productivity of each promotional channel, the specific timeline from LEP ideation to formation, the sociodemographic variability of our applicants, and real LEP members’ feedback on the formation process. We conclude by highlighting what we perceive as keys to success, including collaboration with trusted community partners and a thoughtfully designed interest survey that enabled us to narrow a large applicant pool to a diverse set of invitees. We also describe challenges and lessons learned, including opportunities to better articulate LEP responsibilities during interviews and ways to navigate the tensions inherent in selecting members based on maximum variance. Researchers are encouraged to invest time and resources into building relationships with community partners, knowing that these relationships lay the groundwork for reaching diverse patients and caregivers to advise research.

## Introduction

Patients and caregivers are increasingly recognized as vital to the development of evidence-based practices in clinical care [1]. The benefits of engaging patients and caregivers in any or all phases of a research project are numerous. Research projects conducted in collaboration with patients and caregivers show more sensitive, inclusive, innovative, and rigorous research designs [2-4]; improved enrollment and lower rates of attrition [5,6]; and greater satisfaction among study participants [3]. When research engages patients and caregivers in its design, its priorities become more relevant to the needs of the population as a whole [3]; findings are more meaningful and accessible [3,5]; and any resulting interventions enjoy greater uptake and faster evaluation by regulators and other decision-makers, thereby accelerating their potential for positive impact [3]. Meanwhile, patients and caregivers engaged in research from the development of supportive relationships derive personal meaning from the work and gain professional skills and opportunities [4,7,8].

One mechanism for facilitating engagement with patients and caregivers is through a lived experience panel (LEP). An LEP is an intentionally assembled group of people with relevant personal experience related to the research topic in question [9,10]. LEPs can be engaged in any or all aspects of the research project, such as identifying research priorities [11], reviewing study instruments [10], disseminating recruitment materials [12], interpreting results [10], and providing other input on how studies are conducted and how findings are disseminated [9,11]. LEP activities include, but are not limited to, participating in regular meetings, spending time preparing for meetings, and providing feedback on meeting proceedings, with opportunities for further engagement or learning in between [9,11].

Fully realizing the potential of an LEP to meaningfully inform the research process requires intentionality in LEP composition [13]. LEP composition should align with the range of lived experiences in the population of focus to ensure that the research is maximally relevant [14]. The population of focus could be relatively narrow, such as rural older adults living with chronic heart failure, or a broader population, such as adults living with chronic health conditions. Within a given patient or caregiver population, there are myriad intersecting identities, contextual factors, and experiences that lead to critical differences in the patient or caregiver experience. Even within the more narrowly defined population of rural older adults living with chronic heart failure, variables such as years since diagnosis, gender, race and ethnicity, distance from health care facilities, and caregiver support create distinct experiences that are important to represent in an LEP [15,16]. LEP composition is not only informed by the variation within the population of focus but also by the need to include patients and caregivers who have historically been underrepresented in research and thus excluded from evidence-based care [13,17]. This includes health disparity populations, as well as those with limited technology access, those who are not located near a major academic medical center, and those whose health condition or role in the health system has not been a focus of major federal research funding [18,19].

There are several challenges associated with intentionally composing an LEP with the appropriate variability. Mechanisms to recruit patient or caregiver advisors for research are often based on convenience or pre-existing relationships [9,20]. Assembling an LEP may require researchers to reach beyond the people they already know if they wish to achieve maximum variability [10]. Recruiting “into the unknown” may be overwhelming because of the perceived time commitment, financial expense, and staff effort [5,6]. Furthermore, prioritizing variability raises questions about how to approach potential LEP members, in part on the basis of their unique identities, while steering clear of tokenism [21]. It must be made clear that the research team is not simply trying to meet a diversity requirement or “check a box,” but rather trying to truly integrate a multitude of perspectives to generate more nuanced, responsive, and ethical research [21,22].

In this paper, we present a process for forming an LEP that is grounded in 3 motivating factors: the benefits of engaging patients and caregivers in research, the importance of engaging a maximally variable subset of a given patient population, and the relative absence of explicit processes for achieving these goals [23]. We provide a process for intentionally and respectfully forming an LEP and describe its feasibility.

## Context for LEP Formation

We formed an LEP of US caregivers of adults with chronic health conditions to partner with researchers on a study about how the context of caregiving shapes research engagement. We defined a caregiver as someone—a family member, friend, or other community member—who provides unpaid support for another adult (or adults) with a long-term health condition. The aims of the broader study are to identify and characterize the contextual barriers and facilitators that shape caregiver research engagement and to develop an original, comprehensive measure of the contextual barriers and facilitators to meaningful research engagement.

The team comprised dual principal investigators and research staff, an academic advisory panel (AAP), and community coinvestigators in addition to the 7-member LEP. Table 1 provides a description of each role and the rationale for inclusion. The LEP was established following award notification and was designed to span the full 2-year study period, with panel members participating in quarterly 90-minute meetings. All meetings were virtual. The budget allocated US $500 per meeting for participant stipends to offset lost wages or to pay for care for the care recipient. There was a separate budget for necessary accommodations, including access to technology and interpretation services.

**Table 1.** Function of each project team role and rationale for inclusion.

Role	Rationale	Function	Engagement frequency
Academic advisory panel	Breadth of expertise in designing, conducting, and disseminating community-engaged research Methodological input Access to populations of interest	Advisory role on key decisions	Quarterly
Lived experience panel	Experiential knowledge derived from caregiving responsibilities Increase relevance of project and fit within real-world caregiver context	Advisory role on key decisions	Quarterly
Community coinvestigator	Expertise in primary project content areas, including caregiving and community advocacy Continuous integration of contextual knowledge into decision-making	Collaborator on all decisions	Weekly

## Ethical Considerations

Engaging an LEP was not considered to be human subjects research by the Vanderbilt University Medical Center and University of Virginia Institutional Review Boards because the interested individuals are not potential research participants [24,25]. Rather, they are potential partners in the project.

## Process for Forming the LEP

### Step 1: Laying the Foundation With Academic and Community Partners

#### Community Coinvestigators

Prior to initiating this research project, the principal investigators established relationships with community partners through years of engaged research. From these relationships, the principal investigators engaged 5 partners as coinvestigators on the team for the project while writing the proposal. Community coinvestigators included caregivers and community members with lived experience in community advocacy and in engaging health disparity populations.

#### Academic Advisory Panel

When writing the proposal, the principal investigators identified 5 researchers with experience and academic expertise that complemented those of the academic members of the research team and invited them to join the AAP.

#### Community Organizations

During the grant-writing phase of the project, our team reached out to community organizations to explain the goals of the project and to request their assistance with disseminating materials. Contact with community organizations was established through warm handoffs by community coinvestigators and members of the AAP.

### Step 2: Development of LEP Promotional Materials

The community coinvestigators and AAP members were instrumental in creating promotional materials that would resonate with caregivers while seeking to use understandable and respectful language. We shared a brief description of the LEP that could be copied and pasted into an email to make it easier for others to promote the opportunity. Our team also developed a printable flyer to advertise the opportunity to join the LEP (Multimedia Appendix 1). The flyer was developed in English and Spanish to account for the large population of Spanish-speaking caregivers; it included photos of caregivers of different genders and racial and ethnic identities, specified that all responsibilities were virtual, and indicated that the opportunity was paid, though the amount of compensation was not included [26]. We omitted compensation details to minimize interest from individuals who were primarily financially motivated, including individuals pretending to be caregivers.

### Step 3: Development of the Interest Survey

Our team developed an interest survey to act as an initial screening tool for those wanting to join the LEP (Multimedia Appendix 2). In creating the survey, we sought to balance the competing priorities of gathering information relevant to the caregiver context without creating an undue workload. The first step in creating the interest survey was to identify the primary and secondary variables across which representation was important. These variables were selected through a review of the caregiving literature and full team consensus-building discussions, and included dimensions of sociodemographics, caregiving experience, and prior research engagement experience [26-30]. In addition to these variables, we inquired about applicants’ reasons for their interest in joining the LEP, whether they had engaged in research before, and how the caregiver learned about this opportunity. The full interest survey can be found in Multimedia Appendix 2 and was also available in Spanish.

### Step 4: Distribution of Materials to Share the Opportunity

All team members, including principal investigators, the AAP, and community coinvestigators, were actively engaged in distributing promotional materials. Community organizations and research networks or registries also shared the opportunity. All were asked not to share the survey on social media to prevent fraudulent responses [31]. After the survey had been live for 2 weeks, we reviewed the maximum variance sampling matrix (see “Step 5: Selecting Interviewees Using Maximum Variance Sampling” section) to understand who had expressed interest and identified key gaps in representation. We used this information to determine which additional partners to engage.

### Step 5: Selecting Interviewees Using Maximum Variance Sampling

Our objective was to create an LEP with a broad range of identities and experiences relevant to caregiving for adults with chronic health conditions, positioning the LEP to reflect key dimensions of variability found among US caregivers. To this end, we used a maximum variance sampling approach to identify which applicants to interview [32]. To do this, a matrix was created listing each interested individual by the maximum variance sampling criteria. In this matrix, each column represents an eligible applicant, and each row represents a sampling criterion (eg, relationship to care recipient, US region, and gender). Table 2 provides an example of a maximum variance sampling matrix.

**Table 2.** Example of a maximum variance sampling matrix.

Characteristic	Applicant 1	Applicant 2	Applicant 3
Relationship to care recipient			
Child	✓		
Partner or spouse		✓	
Neighbor			✓
US region			
Northeast	✓		
Midwest		✓	
Southwest			✓
Gender			
Female	✓		
Male		✓	
Nonbinary			✓

We prioritized variability in characteristics that are demonstrated to shape the caregiving experience, while acknowledging that we could not capture every relevant factor [27]. In terms of demographics, we sought variability across race or ethnicity, gender, and geographic location. In terms of caregiver experience, we prioritized variability across caregiver relationships, cohabitation status, and chronic conditions. In terms of research engagement experience, we sought a mix of caregivers with and without research engagement experience. For those with experience, we sought variability in the type and duration of engagement. To the extent possible, we also attended to additional factors in constructing our maximum variance sample, including identities associated with health disparities and other caregiver and engagement experiences.

Because the LEP size was intentionally small, it was not possible to represent every combination of identities and experiences. Rather, applicants with multiple characteristics of interest were prioritized. For example, a research team might identify a potential interviewee who is a female caregiver of a parent living in the Northeast. The team would then look for a caregiver who differed in terms of their gender, relationship to the care recipient, and/or geographic location. This was the process used by 1 principal investigator to review the matrix and make interviewee recommendations based on prioritized criteria to the rest of the team. Team members then reviewed the matrix to suggest additional interview candidates.

### Step 6: Interviewing Potential LEP Members

Each potential LEP member was invited to participate in a 30-minute interview with the dual principal investigators and the project manager. A coinvestigator fluent in Spanish attended the interviews to provide interpretation. Interviewers began by providing background information on the goal of the broader research project and the required and optional responsibilities of the LEP. Interviewees were informed that they might be asked to assist with study activities such as the creation of promotional materials, the development of an interview guide, the analysis of interview data, survey development, and the dissemination of findings. An example question asked of interviewees was as follows: “Can you start out by telling us why you are interested in joining the lived experience panel?” Interviewees were invited to ask any questions or express any concerns. The full interview guide can be found in Multimedia Appendix 3.

### Step 7: Selecting LEP Members

Following each interview, 3 interviewers discussed the applicants’ responses and reviewed their sociodemographic variability as described in the maximum variance sampling matrix. After 8 interviews were completed, the interview team met to discuss an initial set of invitations to join the LEP. Five applicants were selected and emailed invitations to join. The interview team then conducted 3 additional interviews with the goal of inviting 2 additional applicants.

Email invitations indicated our desire for the LEP member to join and included a statement that we would follow up with study onboarding activities, such as setting up payment processes, should they choose to accept our invitation. We also noted that the first LEP meeting would occur approximately 2 weeks from the date of the invitation, so that the LEP could advise on recruitment for the research study, and we noted that future meetings would be scheduled well in advance with consideration of their availability. Prior to the first meeting, LEP members were asked if they would like a Spanish translator present, and all members declined.

## Approach to Evaluation

We evaluated the feasibility of the process using quantitative and qualitative approaches.

### Quantitative Approach

We assessed (1) the time spent forming the LEP, from developing promotional materials to the final formation of the LEP; (2) the success (in terms of the number of applications) of each channel used to reach potential LEP members; and (3) the sociodemographic and caregiving characteristics of those who applied, were interviewed, and were invited to join the LEP.

### Qualitative Approach

We obtained LEP members’ feedback on the LEP formation process through an open-ended question: “From your perspective as a potential LEP member, what felt easy or challenging about recruitment?” Responses were collected from LEP members via email and through discussions at an LEP meeting. We summarize the responses of LEP members to these questions.

## Results

### Time Spent Forming the LEP

**Figure 1.**
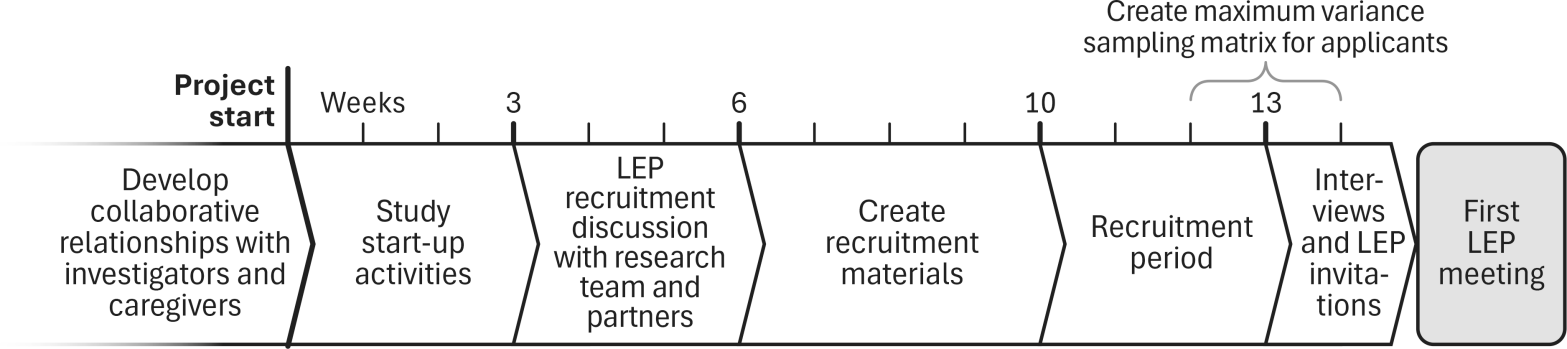
Lived experience panel (LEP) formation timeline.

The entire promotional process, from drafting and finalizing materials to closing the interest survey, spanned from May 2025 to June 2025. The interest survey was published and distributed beginning on June 3, 2025, and remained open for 4 weeks, during which time we received a total of 44 applications to join the LEP. Most applicants who received an interview invitation (10/11) responded on the same day it was sent. All invitees scheduled their interviews for within 8 days of receiving the invitation. All but 1 invited LEP member accepted the invitation to join on the day it was sent. Figure 1 provides a detailed LEP creation timeline.

### Success of LEP Promotional Channels

The LEP opportunity was distributed directly via 12 distinct community coinvestigators, academic advisors, and community partners. Of the 44 applicants, 14 (31.8%) heard about the opportunity through a promotional channel other than our direct partners, signaling that the opportunity was further disseminated through word-of-mouth. Table 3 provides the number of applicants gained through each promotional strategy.

**Table 3.** Lived experience panel applicants gained through each promotional strategy.

Source	Applied (n=44), n (%)	Interviewed (n=11), n (%)	Invited (n=7), n (%)
Community coinvestigator (n=5)	23 (52.3)	3 (27.3)	3 (42.9)
Community organization (n=2)	5 (11.4)	4 (36.4)	2 (28.6)
Academic advisory panel member (n=5)	2 (4.5)	0 (0)	0 (0)
Unspecified email, friend or fellow caregiver, support group, wellness center, and social media account	14 (31.8)	4 (36.4)	2 (28.6)

### Sociodemographic and Caregiving Characteristics of LEP Applicants

We conducted 11 interviews and extended 7 invitations to join the LEP. All participants who were invited to join accepted the invitation. Table 4 provides the demographic and clinical characteristics of those who applied, were interviewed, and were invited to join. It took a median of 23.01 (IQR 37.05) minutes to complete the interest survey.

**Table 4.** Demographic and clinical characteristics of the applicants, interviewees, and invited lived experience panel members.

Demographic or caregiving characteristic	Applied (n=44), n	Interviewed (n=11), n	Invited (n=7), n
Age (y)			
30‐39	4	1	0
40‐49	7	2	1
50‐59	17	5	4
60+	16	3	2
Gender			
Female	35	8	4
Male	8	3	3
Nonbinary	1	0	0
Race and ethnicity^a^			
Asian or Asian American	1	1	1
Black or African American	12	3	2
Hispanic or Latino	2	2	2
White or Caucasian	28	6	3
Not listed: West Indian	1	0	0
I don’t want to answer	2	0	0
LGBTQIA+^b^			
Yes	1	1	0
No	40	10	7
I don’t want to answer	3	0	0
Education			
Finished high school	1	1	1
Some college	5	0	0
Finished 2-year associate’s degree or certificate	8	5	2
Finished a bachelor’s degree at a 4-year college	9	1	0
Finished master’s degree(s)	15	4	4
Finished a PhD, MD, JD, or PharmD	6	0	0
State of residence			
California	2	2	1
Colorado	1	0	0
Indiana	2	1	1
Illinois	9	1	1
Michigan	3	2	2
Georgia	2	1	1
New Jersey	1	0	0
New York	2	0	0
North Carolina	1	1	0
Ohio	1	0	0
Pennsylvania	1	1	1
South Carolina	1	0	0
South Dakota	15	2	0
Wisconsin	3	0	0
Geographic region			
Rural	9	2	1
Suburban	15	3	2
Urban	17	6	4
I move between types of places	2	0	0
Not listed: midsized city	1	0	0
Employment outside of unpaid caregiving^a^			
Looking for work	4	3	3
Working ˂30 hour/week	9	1	0
Working ≥30 hour/week	17	4	2
Student (full or part time)	2	2	1
Retired	9	1	1
Not working	3	0	0
Other (“adjunct professor”)	1	0	1
I don’t want to answer	2	0	0
Experience in research			
Yes	20	6	4
No	23	5	3
Relationship to care recipient^c^			
Nonrelative	9	2	2
Sibling	3	0	0
Partner or spouse	13	2	1
Parent or parent-in-law	22	6	4
Other relative	7	3	2
Years spent caregiving			
<1	1	1	0
≥1 and ˂10	29	8	5
≥10	14	2	2
Top 10 care recipient condition^a^^,^^c^			
Alzheimer disease and related dementias or cognitive decline	33	7	4
Diabetes mellitus (type 1 or 2)	12	4	4
Heart failure, cardiac issues, or atrial fibrillation	10	5	5
Kidney disease, renal disorder, or low function	10	2	2
Parkinson disease	5	1	1
Stroke (including paralysis, recovery, and aphasia)	6	2	2
Arthritis, osteoarthritis, or rheumatoid arthritis	6	2	1
High blood pressure	7	1	1
Cancer (prostate, lung, ovarian, brain, and pancreas)	5	2	2
Chronic obstructive pulmonary disease	3	1	1
Time spent caregiving^c^			
A little time	4	1	1
A fair amount of time	8	2	1
A significant amount of time	14	4	2
A lot of time	10	2	2
I provide 24-hour support	18	4	3
Frequency of contact with care recipient^c^			
They live with me	24	5	3
I see them daily	5	2	2
I see them a few times a week	8	3	2
I provide support from a distance	4	1	1
Other	13	2	1

a Participants could select more than 1 response option.

b LGBTQIA+: lesbian, gay, bisexual, transgender/transsexual, queer, and other minority sexual orientations and gender identities.

c Participants who cared for 2 people selected 2 or more response options.

### LEP Member Feedback on the Formation Process

Five LEP members provided feedback by email, and 2 of them expanded on their comments during a subsequent live LEP discussion.

#### Overall Process

In response to our request for feedback on the steps to joining the LEP, 1 LEP member reported that it “was done in such a way as to help applicants feel valued (choice of words, tone, organized process, individual interview, invitation to contact researchers with questions)” (LEP1). Another LEP member described the LEP formation as a “smooth process” that did not overwhelm him, “I’m busy, working full time, caregiver and stuff, so it had to be easy enough that I could find a time to do it” (LEP5). Two LEP members (LEP1 and LEP2) shared that they wished there had been more concrete information in the interview about the “number of hours or type of responsibilities/work” associated with joining the LEP (LEP2). One wrote, “I haven’t always been clear on what is expected of me as an LEP member, especially given the generous compensation... I would simply feel more comfortable with a general guideline, such as expecting about 1 request per month” (LEP1).

#### Interest Survey

According to 1 LEP member, the interest survey questions “felt meaningful, not just like a checklist” and helped her to “reflect on why my voice and story could be helpful” (LEP4). In a group discussion, another LEP member noted that the questions in the interest survey seemed to have a clear rationale, noting that this is not always the case in research engagement (LEP1). After submitting the interest survey, 1 LEP member said it was challenging when the research team did not confirm its receipt (LEP2).

#### Interviews

Regarding the interview process, 1 LEP member found this to be the most challenging part, stating, “Not knowing if I was the right person for the job” (LEP3). One member indicated that “The interview was good as I got to ask a number of questions about the aim of the LEP, the [redacted] study, and the kind of research that was being undertaken” (LEP2). Another member used the interview to “decide if this is a safe space” (LEP4). According to this LEP member, those conducting the interview “leveled” the power dynamic and created a “peer-to-peer connection” by sharing their own perspectives and admitting that they did not have all the answers. These small steps signaled to the interviewee that “this was a space where real experiences mattered” (LEP4).

#### Deciding to Join

One LEP member reported that the decision to join was challenging given the time constraints associated with caregiving. This member noted, “I wanted to be sure my time, my story, and my emotional labor would be respected as well” (LEP4). Ultimately, she joined because she believed that “my presence could genuinely make an impact” (LEP4). For another LEP member, the decision to join was easy. He surmised that “most people who embrace their role as a caregiver are more than eager to participate and get involved” (LEP5). A third LEP member provided their rationale for joining the LEP, explaining that it “could somehow help future generations” and that he saw it “as a learning experience, contributing and also receiving input from others in similar situations” (LEP3).

## Discussion

### Summary

In this study, we describe a process for intentionally and respectfully forming an LEP and evaluate its feasibility. We worked with a broad range of partners to form an LEP of 7 caregivers, previously unknown to the principal investigators. Moreover, the resulting LEP included caregivers with a broad range of characteristics aligned with the diverse lived experiences of the US caregiver population [26]. Feedback from LEP members indicated that while some experienced the process of joining as relatively smooth, others identified areas for improvement. LEP members’ decisions to join were shaped by their experiences at each step, from hearing about the opportunity to completing the interest survey to engaging in the interview. With refinements to better support applicants, the process for forming an LEP described here can be adopted to facilitate research engagement across multiple patient and caregiver populations.

### Key Takeaways

We identified 2 key takeaways from our process of forming an LEP. First, we found success in promoting the LEP opportunity through our trusted community partners. Relationships with these partners allowed us to quickly form an LEP that is diverse in the characteristics that shape the caregiving experience. We recommend that research teams focused on building variable LEPs prioritize first building relationships with community members. As a second takeaway, we were rewarded for our strategy of intentionally selecting questions for the LEP interest survey in ways we did not predict. Not only did the selected questions allow us to rapidly narrow a pool from 44 applicants to 11 interviewees to 7 invitations to join, but the questions served a second purpose of building trust with applicants, with 1 LEP member describing the survey questions as well-justified and meaningful. We recommend that research teams invest time and thought into crafting an interest survey that provides actionable information for researchers and communicates respect to applicants.

### Lessons Learned

In addition to our key takeaways, we learned 3 lessons that will shape how our team forms LEPs going forward. First, with input from our LEP members, we learned that our interview process could have more clearly conveyed the expectations and responsibilities of LEP membership. LEP members’ desire for specificity raises an important question for future work: how to offer concrete information about engagement responsibilities while allowing LEP members to shape the nature of their involvement. One approach is to introduce an LEP charter during the interviews—a document describing the scope and commitment of LEP engagement. During interviews, we would describe a formal process for revising the charter so that it is continuously responsive to LEP members’ needs and goals. For example, a group charter could address attendance expectations, alternatives to attendance if meeting as a group is not possible or preferred, processes for member withdrawal, and approaches to facilitating balanced participation between LEP members. The introduction of a charter and its collaborative codevelopment could serve the equally important purpose of building trust with potential LEP members.

Second, the maximum variance sampling approach introduces a central tension: decisions made to maximize variability may inadvertently imply to nonselected applicants that the researchers are not interested in their experiences. This tension felt especially acute when variability was the key reason for not inviting an interviewed applicant, as we felt connected to the interviewees and knew more about the depth and nuance of their experiences. In future applications of the maximum variance sampling approach, we will emphasize throughout the interest survey and interview process that all caregiving experiences are meaningful, even though the limited size of LEPs means not all caregivers can be invited to join. We will explicitly state that our selection decisions prioritize variability, not because it is the only relevant guiding principle, but because diverse LEPs strengthen the relevance and impact of the research they inform [33,34]. As another attempt to respect applicants’ time and contributions, in future iterations of LEP formation, we will interview and accept applicants sequentially rather than in batches so that we do not interview many more people than we can invite to join.

Finally, although we were successful in disseminating the LEP opportunity to a diverse community of caregivers, certain gaps in our applicant pool persisted. For example, we heard from few caregivers who identified as Latino, who had less than a high school education, who identified as LGBTQIA+ (lesbian, gay, bisexual, transgender/transsexual, queer, and other minority sexual orientations and gender identities), or who lived in the southwestern region of the United States. These gaps highlight the need for our team to leverage different promotional strategies (eg, posting flyers in-person rather than relying on digital dissemination) and to further expand and diversify our community partnerships.

### Conclusions

The findings of this paper bring to light a second, central tension: engaging patients and caregivers requires a significant, and not directly compensated, investment of time and resources. Our formation of an LEP hinged on existing relationships with community partners, in which trust was sufficient for them to agree to be coinvestigators or assist with the dissemination of LEP promotional materials. To those intimidated by this proposed groundwork, we offer 3 forms of encouragement. First, researchers new to patient and caregiver engagement can reach out to colleagues and mentors with long-standing relationships in communities of interest to help accelerate the building of trust. Second, investments in community relationships do not signify a net loss of time. Instead, these investments are reallocations of time toward earlier phases of the work, increasing our productivity downstream in meeting LEP formation milestones and the successful dissemination of materials. Finally, there are undoubtedly additional, innovative strategies for reaching diverse and previously unknown patients and caregivers, and teams that blend research and community perspectives are more likely to uncover them. These maximally variable LEPs, comprising people embedded in patient and caregiver communities, can substantially enhance the relevance and impact of any research they support.

## Supplementary material

10.2196/95145 Multimedia Appendix 1 Promotional flyers to join the lived experience panel.

10.2196/95145 Multimedia Appendix 2 Interest survey to join the lived experience panel.

10.2196/95145 Multimedia Appendix 3 Lived experience panel interview questions.

## Abbreviations

AAP: academic advisory panel
LEP: lived experience panel
LGBTQIA+: lesbian, gay, bisexual, transgender/transsexual, queer, and other minority sexual orientations and gender identities

## References

[1] Forsythe LP, Carman KL, Szydlowski V (2019). Patient engagement in research: early findings from the Patient-Centered Outcomes Research Institute. Health Aff (Millwood).

[2] (2004). Community-based participatory research: assessing the evidence: AHRQ evidence report summaries. https://www.ncbi.nlm.nih.gov/sites/books/NBK11852/.

[3] Vat LE, Finlay T, Jan Schuitmaker-Warnaar T (2020). Evaluating the “return on patient engagement initiatives” in medicines research and development: a literature review. Health Expect.

[4] Béland S, Lambert M, Delahunty-Pike A (2022). Patient and researcher experiences of patient engagement in primary care health care research: a participatory qualitative study. Health Expect.

[5] Domecq JP, Prutsky G, Elraiyah T (2014). Patient engagement in research: a systematic review. BMC Health Serv Res.

[6] Levitan B, Getz K, Eisenstein EL (2018). Assessing the financial value of patient engagement: a quantitative approach from CTTI’s Patient Groups and Clinical Trials Project. Ther Innov Regul Sci.

[7] Jolliff A, Holden RJ, Valdez R (2024). Investigating the best practices for engagement in remote participatory design: mixed methods analysis of 4 remote studies with family caregivers. J Med Internet Res.

[8] Lachance L, Coombe CM, Brush BL (2022). Understanding the benefit-cost relationship in long-standing Community-based Participatory Research (CBPR) Partnerships: findings from the Measurement Approaches to Partnership Success (MAPS) Study. J Appl Behav Sci.

[9] Ahmed M, McLean J, Donaldson C, Roy MJ, Baker R (2025). Creating the conditions for meaningful and effective PPIE in community-based public health research: learning from a UK-wide lived experience panel. Res Involv Engagem.

[10] McClelland H, O’Connor RC, Gibson L, MacIntyre DJ (2025). Exploring web-based support for suicidal ideation in the Scottish population: usability study. JMIR Form Res.

[11] Bennett AV, O’Brien K, Moreno M (2024). Development of a lived experience panel to inform the design of embedded pragmatic trials of dementia care interventions. J Am Geriatr Soc.

[12] Gazaway S, Bakitas M, Underwood F (2023). Community informed recruitment: a promising method to enhance clinical trial participation. J Pain Symptom Manage.

[13] Thomson R, Murtagh M, Khaw FM (2005). Tensions in public health policy: patient engagement, evidence-based public health and health inequalities. Qual Saf Health Care.

[14] (2023). Strengthening the diversity and role of patient and family advisory councils: opportunities for action. https://ipfcc.org/bestpractices/patient-and-family-advisory-programs/IPFCC_Strengthening_Diversity.pdf.

[15] Coats AJS (2019). Ageing, demographics, and heart failure. Eur Heart J Suppl.

[16] Mohammed AS, Takagi MA, Yasmeen U (2025). Nationwide trends in demographics, comorbidities, and mortality among elderly patients with heart failure with preserved ejection fraction hospitalized with cardiac arrest. J Pers Med.

[17] Green CL, Aladesanmi O, Chery G, Jackson 2nd LR, Thomas KL (2026). Racial representation in heart failure clinical trials and registries: a systematic review and meta-analysis. J Natl Med Assoc.

[18] Oldhoff-Nuijsink C, Fransen MP, Peute LW, Derksen ME (2025). Strategies for engaging “hard-to-reach” populations in a panel for digital health research: a qualitative study among experts. PLOS Digit Health.

[19] Our biggest health challenges. National Institutes of Health (NIH).

[20] Yuan NP, Mayer BM, Joshweseoma L, Clichee D, Teufel-Shone NI (2020). Development of guidelines to improve the effectiveness of community advisory boards in health research. Prog Community Health Partnersh.

[21] Hahn DL, Hoffmann AE, Felzien M, LeMaster JW, Xu J, Fagnan LJ (2017). Tokenism in patient engagement. Fam Pract.

[22] Majid U (2020). The dimensions of tokenism in patient and family engagement: a concept analysis of the literature. J Patient Exp.

[23] Gilfoyle M, Melro C, Koskinas E, Salsberg J (2023). Recruitment of patients, carers and members of the public to advisory boards, groups and panels in public and patient involved health research: a scoping review. BMJ Open.

[24] (2000). HRPP I.B—activities subject to IRB jurisdiction. https://public.powerdms.com/VanderbiltUMC/tree/documents/2355652.

[25] (2025). Standard operating procedures (SOP). https://hrpp.research.virginia.edu/media/826.

[26] (2025). Caregiving in the US report 2025. https://www.caregivingintheus.org/reports/caregiving-in-the-us-report-2025/.

[27] (2025). Caregiving in the US 2025: caring across states. https://www.aarp.org/content/dam/aarp/ppi/topics/ltss/family-caregiving/cgus-2025-caring-across-states/caregiving-in-the-us-2025-caring-across-states.doi.10.26419-2fppi.00383.001.pdf.

[28] Adelman RD, Tmanova LL, Delgado D, Dion S, Lachs MS (2014). Caregiver burden: a clinical review. JAMA.

[29] Shuffler J, Lee K, Fields N, Graaf G, Cassidy J (2023). Challenges experienced by rural informal caregivers of older adults in the united states: a scoping review. J Evid Based Soc Work.

[30] Choi JY, Lee SH, Yu S (2024). Exploring factors influencing caregiver burden: a systematic review of family caregivers of older adults with chronic illness in local communities. Healthcare (Basel).

[31] Pozzar R, Hammer MJ, Underhill-Blazey M (2020). Threats of bots and other bad actors to data quality following research participant recruitment through social media: cross-sectional questionnaire. J Med Internet Res.

[32] Patton MQ (2014). Qualitative Research & Evaluation Methods: Integrating Theory and Practice.

[33] Meinertz NR, Lee J, Kim Y (2025). Are family caregiving programs appropriate for minority caregivers? A systematic review of 37 caregiving programs. J Appl Gerontol.

[34] Yellow Horse AJ, Patterson SE (2022). Greater inclusion of Asian Americans in aging research on family caregiving for better understanding of racial health inequities. Gerontologist.

