# Supplementary figures and images for: Using Partner-Driven Maximum Variance Sampling to Form a Lived Experience Panel: Step-by-Step Tutorial
Source: J Particip Med. 2026 Jun 26;18:e95145. doi: 10.2196/95145 (PMC13308908; doi:10.2196/95145)

Appendix A. Promotional Flyers to Join the Lived Experience Panel


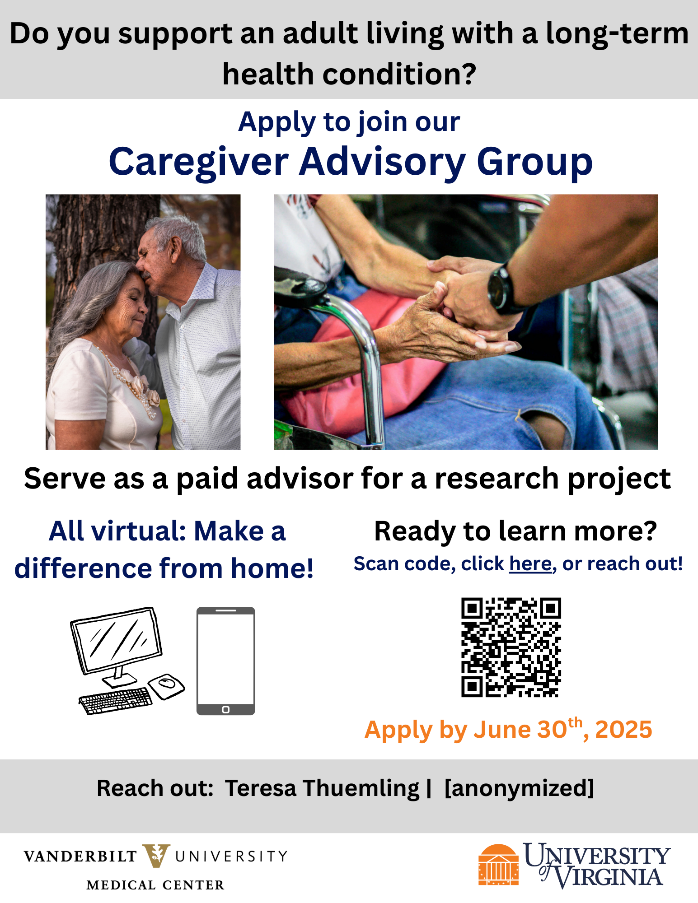


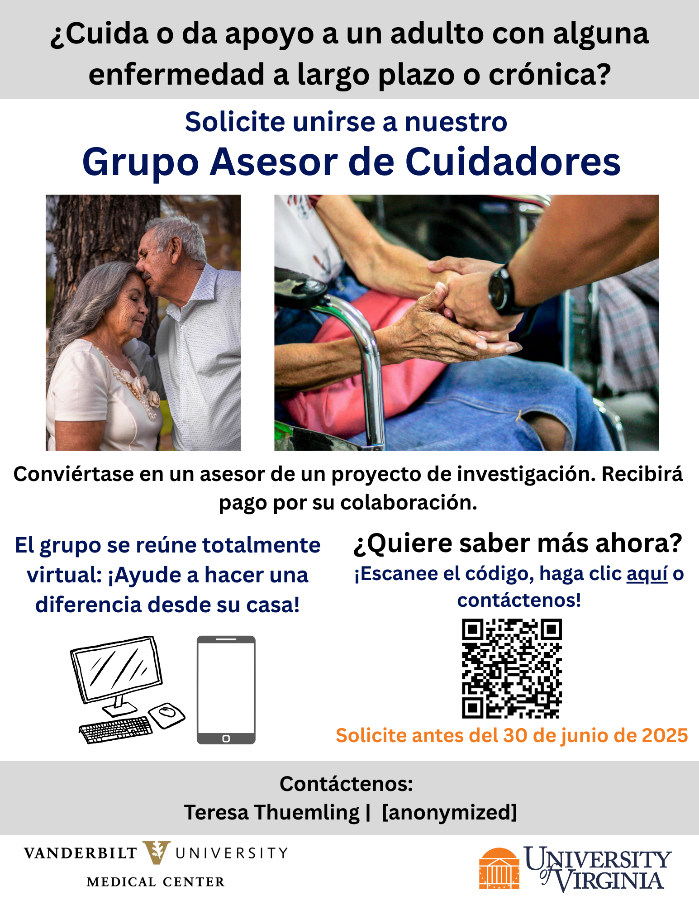

Supplement: Multimedia Appendix 1 [file jopm-v18-e95145-s001.docx]
