# Supplementary material for: Using Partner-Driven Maximum Variance Sampling to Form a Lived Experience Panel: Step-by-Step Tutorial
Source: J Particip Med. 2026 Jun 26;18:e95145. doi: 10.2196/95145 (PMC13308908; doi:10.2196/95145)
Supplement: Multimedia Appendix 2 [file jopm-v18-e95145-s002.docx]

Appendix 2. Interest Survey to join the LEP

In this survey, we will ask you why you want to join the Lived Experience Panel. We will also ask some questions about you and the person you care for or used to care for.  We are asking these questions because we want to bring together people with many different experiences. You can skip any question that you do not want to answer. If we think you might be a good fit for the Lived Experience Panel, we will call or email you to schedule a time to talk further.

**Eligibility Criteria**

1. Do you currently live in the United States?

Yes

No [directs to end of survey]

1. Are you at least 18 years old?

Yes

No [directs to end of survey]

1. Do you currently care for an adult (18 years or older) with a long-term health condition, or have cared for an adult with a long-term health condition in the last two years?

Yes

No [directs to end of survey]

1. What types of help or care do you give, or have given, to the person with a long-term health condition(s)?  For example, making meals, driving them places, giving them their medications, helping them take a shower or use the restroom, helping with financial, legal, and healthcare matters, listening to and spending time with them, and more.

[free text]

1. Are you the person (or one of the people) who provides most of the support to this adult?

Yes

No

**Interest Questions**

1. Why are you interested in joining the Lived Experience Panel?

[free text]

1. How did you hear about this opportunity?

 [free text]

1. Have you participated or wanted to participate in research before? Tell us about the research.

[free text]

**Availability Questions**

1. If you are a part the Lived Experience Panel, you will need to join four 90-minute meetings each year. The first meeting will be at the beginning of July.  When could you make time for the July Lived Experience Panel meeting? Please mark “yes” or “no” for each weekday and time of day in the table below.  Remember, all meetings will be on Zoom and you can also call in.

|  | Morning | Afternoon | Evening |
| --- | --- | --- | --- |
| Mondays | Y/N |  |  |
| Tuesdays |  |  |  |
| Wednesdays |  |  |  |
| Thursdays |  |  |  |
| Fridays |  |  |  |

1. What time zone do you live in?
2. Eastern Daylight Time
3. Central Daylight Time
4. Mountain Daylight Time
5. Mountain Standard Time
6. Pacific Daylight Time
7. Alaska Daylight Time
8. Hawaii-Aleutian Standard Time

**Caregiving Information**

1. How many adults with long-term health conditions do you care for, or have you cared for, in the past two years?

1 [proceed through questions]

2 [need to ask to ask questions for each of the number indicated]

3 [need to ask to ask questions for each of the number indicated]

4+[need to ask to ask questions for each of the number indicated]

[IF >1 “Since you have cared for more than one person, we’d like to ask about each of those people.”]

[If >1 "Think about the first person....etc.]

1. Are you currently caring for this person?
2. Yes
3. No
4. What is your relationship to this person?
5. Partner or spouse
6. Parent or parent-in-law
7. Grandparent
8. Sibling
9. Other relative [free text]
10. Nonrelative [free text]
11. For how long have you been a caregiver for this person?
12. Less than one year
13. _______ years [numeric answers only]
14. What long-term health condition(s) does this person have?

[free text]

1. How much time do you spend caring for this person?
2. A little time
3. A fair amount of time
4. A significant amount of time
5. A lot of time
6. I provide 24-hour support
7. How often do you see the person you care for?
8. They live with me
9. I see them daily
10. I see them a few times a week
11. I provide support from a distance
12. Something else [free text]
13. Where does this person live?
14. The person lives with me
15. A private home, apartment, or similar residential setting
16. An independent living or retirement community
17. An assisted living facility where some care may be provided
18. A nursing care or long-term care facility
19. A type of residence not listed here. Describe. [free text]
20. Do any other people help care for this person?

Yes

No

**Demographic Information**

We have just a few more questions, which are about you.

1. How old are you?

[numeric answers accepted] *years old*

1. Which gender best describes you?
2. Man
3. Woman
4. Non-binary
5. Prefer to provide own description [free text]
6. I don’t want to answer
7. Which of these best describes you?  You can select more than one.
8. Asian or Asian-American
9. Black or African American
10. Hispanic or Latino
11. Middle Eastern or North African
12. Native American or Alaskan Native
13. Pacific Islander/Native Hawaiian
14. White or Caucasian
15. Category not listed. Please describe: [free text]
16. I don’t know
17. I don’t want to answer
18. Are you a member of the LGBTQIA+ community?
19. Yes
20. No
21. I don’t know
22. I don’t want to answer
23. Which of the following describes you? You can select more than one.  We are asking about paid work outside of your caregiving role.
24. Working 30 hours a week or more
25. Working less than 40 hours a week
26. Looking for work
27. Part-time student
28. Full-time student
29. Retired
30. Category not listed: [free text]
31. I don’t want to answer
32. How much school have you gone to?
33. No high school
34. Some high school
35. Finished high school
36. Some college
37. Finished an associate’s degree at a 2-year college or certificate in career/technical education
38. Finished a bachelor’s degree at a 4-year college
39. Some graduate school
40. Finished a master’s degree
41. Finished a PhD, MD, or JD
42. Something else. Describe: [free text]
43. I don’t want to answer
44. What best describes where you live?
45. A private home, apartment, or similar residential setting
46. An independent living or retirement community
47. An assisted living facility where some care may be provided
48. A nursing care or long-term care facility
49. A type of residence not listed here. Describe: [free text]
50. In what state do you live?

[all states – drop down]

1. How would you describe the area where you live?
2. Urban
3. Suburban
4. Rural
5. I move between types of places
6. I don’t want to answer
7. Other [free text]
8. What is your zip code?

[zip codes only]

1. Including you, how many people 18 years or older live in your household? [numeric answers only]
2. How many people under 18 live in your household? Please include children who live with you part of the time.

[numeric answers only]

1. How much money did your household make last year before taxes?
2. Under $20,000
3. $20,000 to under $50,000
4. $50,000 to under $100,000
5. $100,000 to under $200,000
6. $200,000 or more
7. I don’t know
8. I don’t want to answer
9. What is your household’s net worth?
10. We owe more money than we have
11. Under $10,000
12. $10,000 to under $50,000
13. $50,000 to under $100,000
14. $100,000 to under $500,000
15. $500,000 to under $1,000,000
16. Over $1,000,000
17. I don’t know
18. I don’t want to answer

**Contact Information**

1. How can we follow up with you about joining the Lived Experience Panel? *You can select more than one.*
2. Phone
3. Email
4. [IF PHONE SELECTED] What is your phone number? [phone numbers only]
5. Can we call you?
   1. Yes
   2. No
6. Can we text you?
   1. Yes
   2. No
7. Can we leave you a voicemail?
   1. Yes
   2. No
8. [IF EMAIL SELECTED] What is your email address?

[email addresses only}

1. What is your preferred language?

[write in]

**Thank you!**

Thank you for taking this survey and for your interest in joining the Lived Experience Panel. A member of the research team will reach out to you with next steps. In the meantime, if you have any questions, please contact [anonymized], a member of our research team.

Email address: [anonymized]

Phone number: [anonymized]
