# Supplementary material for: Using Partner-Driven Maximum Variance Sampling to Form a Lived Experience Panel: Step-by-Step Tutorial
Source: J Particip Med. 2026 Jun 26;18:e95145. doi: 10.2196/95145 (PMC13308908; doi:10.2196/95145)
Supplement: Multimedia Appendix 3 [file jopm-v18-e95145-s003.docx]

Appendix 3

**Lived Experience Panel Interview Questions**

- Hello. Thank you for joining our call. We’re really excited to talk to you about the possibility of joining our advisory group, which is called the Lived Experience Panel (or LEP for short).
- Just as a brief reminder, the goal of our project is to make it easier for people who support an adult living with a long-term health condition to engage in research. Our plan is to create a survey to help better understand what challenges caregivers face when trying to engage in research.
- The Lived Experience Panel will be a group of seven people who each support an adult living with a long-term health condition. This group will meet online and share their ideas to improve our research project. Each person will share their ideas to help make the project better. [Motivation – also gets at caregiving experience]
- Can you start out by telling us why you are interested in joining the lived experience panel?
- Can you tell us about any prior experience engaging in research?
- As a reminder, The LEP will meet over Zoom or phone four times each year for two years – so that’s every 3 months. Each meeting will last an hour and a half with a break in the middle.
  - Do you have concerns about participating in the LEP?
- We also want to mention you will be compensated for your time - you will receive $500 for each LEP meeting ($4,000 total for 8 quarterly meetings over 2 years)
- What other questions or concerns do you have?
